# A Kriging Surrogate Model for Uncertainty Analysis of Graphene Based on a Finite Element Method

**DOI:** 10.3390/ijms20092355

**Published:** 2019-05-13

**Authors:** Jiajia Shi, Liu Chu, Robin Braun

**Affiliations:** 1 School of Transportation and Civil Engineering, Nantong University, Nantong 226019, China; shijj@ntu.edu.cn; 2 Faculty of Engineering and Information Technology, University of Technology, Sydney, Ultimo, NSW 2007, Australia; robin.braun@uts.edu.au

**Keywords:** Kriging surrogate model, graphene sheets, Latin hypercube sampling, finite element method

## Abstract

Due to the inevitable presence of random defects, unpredictable grain boundaries in macroscopic samples, stress concentration at clamping points, and unknown load distribution in the investigation of graphene sheets, uncertainties are crucial and challenging issues that require more exploration. The application of the Kriging surrogate model in vibration analysis of graphene sheets is proposed in this study. The Latin hypercube sampling method effectively propagates the uncertainties in geometrical and material properties of the finite element model. The accuracy and convergence of the Kriging surrogate model are confirmed by a comparison with the reported references. The uncertainty analysis for both Zigzag and Armchair graphene sheets are compared and discussed.

## 1. Introduction

With a two-dimensional (2D) honeycomb lattice, graphene can be wrapped up into zero-dimensional (0D) fullerenes, rolled into one-dimensional (1D) nanotubes, or stacked into three dimesnional (3D) graphite [1]. Graphene has extrodinary properties in potential applications. The electrical conduction of graphene is proved possible and is discovered in the mechanical exfoliation samples [2,3,4]. The measurements of thermal properties of graphene [5,6,7] also confirm the distinguished thermal conductivity of graphene, and, therefore, ignite strong interests of researchers. The unexpected mechanical properties of graphene are experimentally verified through nano-indentation by the atomic force microscope (AFM) [8].

It is recognized in academia that graphene has the astonishing strength and stiffness when compared with the traditional materials. However, the precise values of the parameters corresponding to the material properties of graphene are difficult to determine. On the aspects of experimental measurements, the in-plane Young’s modulus of bulk graphite [9] is in the range of 1.02 ± 0.03 TPa. In the tensile test [10], a broad range of stiffness values (0.27 TPa to 1.47 TPa) were obtained, with breaking strengths ranging from 3.6 to 63 GPa. In addition, the defects in the graphene contribute to the deviation in the bending rigidity in the test results of suspended monolayer graphene membranes [11]. Furthermore, even the same test method is used in the measurements. The results obtained by researchers are different. For example, the Young’s modulus is extracted as 0.5 TPa [12] in the measurement of the bending stiffness of graphene sheets by AFM nano-indentation. In another study, the Young’s modulus equals 1.0 ± 0.1 TPa and the corresponding intrinsic stress is 130 ± 10 GPa at a strain of 0.25 [8].

The fluctuation and deviation in the parameters related with the material properties of graphene are due to the inevitable uncertainties. On the one hand, the nanoscale size in graphene ensures the exact measurement become challenging, and the relative errors caused by equipment or other stochastic factors are amplified and significantly affects the final results. On the other hand, the mathematical expressions and related knowledge in macro scale are limited and not appropriate to describe the physical relationships in microscope. The definition and evaluation of certain parameters in micro scale are required to be developed with accurate recognition.

Different from epistemic uncertainties, the aleatory uncertainties in graphene are also unavoidable in the real situations. First, the random distributed defects contribute to the variation of material properties in graphene [13]. Second, the grain boundaries in macroscopic samples are hard to control. The mechanical parameters are sensitive to the size and shape of grain boundaries of graphene sheets. Third, uncertainties in the sample geometry, stress concentration at clamping points, and unknown load distributions are all the uncertian problems confronted by researchers. Therefore, it is necessary to develop the feasible model, which takes the uncertainties into consideration for the analysis of graphene.

Development of sophisticated models to propagate the uncertainties in the deterministic models are vital issues in the mechanical analysis of graphene. The Latin hypercube sampling (LHS) method is one of the advanced Monte Carlo simulations (MCS). The MCS has been applied in the investigation of the phase transition and magnetism of graphene sheets [14,15,16,17]. When the number of sampling is sufficient, it can reach a good accuracy in numerical computations. It is common to take the results of the MCS as an exact solution or comparison standard [18,19]. By dividing the sample spaces into subspaces, the LHS method effectively avoids the situation of point clustering together and repeating in the MCS [20].

The Kriging surrogate model (KSM) is an interpolation method, which finds its roots in geo-statistics [21,22]. With applications in the design of computer experiments [23], Bayesian prediction is used in the deterministic functions. Furthermore, the global optimization is efficient in the expensive black-box functions [24]. Martin and Simpson [25] discussed the application of KSM to approximate deterministic models. Kleijnen [26] wrote the review to conclude the Kriging meta-modeling in simulation. Wu [27,28] used the Kriging model in the inverse uncertainty quantification of nuclear reactor simulators under the Bayesian framework. Cressie [29] explored the interpolation of spatial data for KSM. In addition, the introduction about KSM in statistics for spatial data were also published [30]. Moreover, Forrester [31] and Roustant [32] developed the Kriging surrogate-based optimization. As one of the most promising spatial correlation models, the Kriging model is more flexible than the regression model and not as complicated and time consuming as other meta-models [33]. The Kriging model is attractive for its prediction accuracy and time saving for the complicated analysis.

This paper proposes the application of the KSM to represent the uncertain and complicated relationship between the elastic response of graphene sheets and the external forces. The LHS method is combined with the finite element model to successfully propagate the uncertainties in parameters corresponding to material and geometrical properties. The accuracy and convergence are confirmed by comparison with the reported references. The uncertainty analysis of Zigzag and Armchair graphene sheets in free vibration is completed and discussed.

## 2. Model Formation

### 2.1. Graphene Sheets

The modified Morse potential is successfully employed to simulate the nonlinear response of nanomaterials under tensile and torsional loading conditions [34]. The effects of defects on the Young’s modulus are studied by the modified Morse potential [35]. In a comparative study, the modified Morse potential provides more accurate predictions of tensile strength and fracture strain for carbon nanotubes than the reactive empirical bond order potential [36,37]. However, in this study, the exact values of the material and geometrical parameters are represented by the corresponding interval ranges as shown in Table 1, according to the related References [38,39,40,41,42,43,44,45,46].

**Figure 1:**
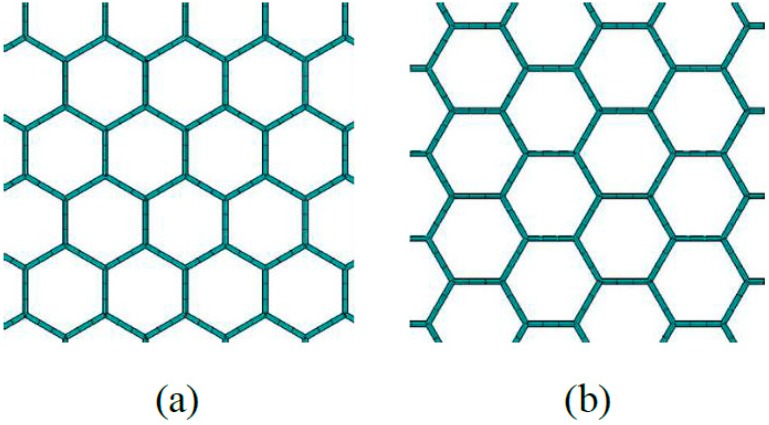
Graphene sheets with beam finite elements (**a**) is for the Zigzag type and (**b**) is for the Armchair type.

In the finite element model of graphene sheets, the length of the C-C bond corresponds to the length of the beam in the planar-frame structure, while the wall thickness is related to the diameter of the cross section in the beam elements. Figure 1 illustrates a schematic diagram of mono-layer graphene in two dimensions. The Zigzag and Armchair types are both included. The beam elements applied in graphene sheets are based on the Timoshenko beam theory, as shown in the previous work [47].

However, the KSM prosposed in this study for uncertainty analysis of graphene based on the finite element method is different with the Monte Carlo based finite element method in the previous work. First, the motivation of KSM is for the uncertainty analysis of pristine graphene. While the Monte Carlo-based finite element method (MC-FEM) is to propagate the vacancy defects in the pristine graphene. Second, the research objects are different. The MC-FEM is more concentrated in the geometrical uncertainties such as vacancy defects. The study in this paper is more comprehensive to explore the uncertainties in the material and geometrical parameters. Third, compared with MC-FEM, the KSM in this paper has the following advantages: compatibility to the experimental data, convenience to the reliable prediction, and good performance in vibration analysis and uncertainty analysis for graphene. The MC-FEM is an effective numerical simulation to take the randomly distributed vacancy defects into consideration. However, the MC-FEM is not compatible to the experimental data or act as a surrogate model to represent the relationships between the input materials and geometrical parameters with the output resonant frequencies as well as their variance and fluctuations. Lastly, besides the vacancy defects in the graphene, the uncertainties and fluctuations in the material and geometrical parameters are also important issues to deal. The work in this study is a crucial supplement for the mechanical research of graphene.

### 2.2. Kriging Surrogate Model

The mathematical description of the KSM is written as the following equation.

(1) $$y(x)=\sum_{j=1}^{n}\beta_{j}f_{j}(x)+z(x)=f^{T}(x)\beta +z(x)$$

where y(x) is the prediction result at a general point x. The first component is a linear regression of the data, the function set $f={\left[f_{1},f_{2},\cdots ,f_{n}\right]}^{T}$ and the vector $\beta ={\left[\beta_{1},\beta_{2},\cdots ,\beta_{n}\right]}^{T}$ as the regression coefficients are included. The introduction of the second component z(x) makes the classical linear regression model become a stationary Gaussian random process model with zero mean and covariance [26].

(2) $$Cov\left[z(x^{i}),\;z(x^{j})\right]=\sigma^{2}R(x^{i},\;x^{j})$$

where $\sigma^{2}$ is the process variance of the KSM and R(⋅,⋅) is the correlation kernel. The valid correlation kernel $R(x^{i},\;x^{j})$ satisfies two conditions: first, the input domain is symmetrical and, second, the resulting correlation is a positive semi-definite matrix.

(3) $$R(x^{i},\;x^{j})=R(x^{j},\;x^{i})$$

The correlation kernel is usually described by a function of the distance.

(4) $$R(x^{i},\;x^{j})=R\left[d(x^{i},\;x^{j})\right]$$

The weighted distance is often written as the following formula.

(5) $$d(x^{i},\;x^{j})=\sum_{k=1}^{d}\frac{{\left|x_{k}^{i}-x_{k}^{j}\right|}^{p_{k}}}{\theta_{k}}$$

Then, if the correlation kernel R(⋅,⋅) is the power-exponential function, the covariance is computed as the equation below.

(6) $$Cov\left[z(x^{i}),\;z(x^{j})\right]=\sigma^{2}\cdot \exp\left[-\sum_{k=1}^{d}\frac{{\left|x_{k}^{i}-x_{k}^{j}\right|}^{p_{k}}}{\theta_{k}}\right]$$

The correlation kernel is important to the performance of the KSM model. The common spatial correlation kernels are the Linear, Exponential, Gaussian, and Power-exponential [26]. Among them, the Gaussian kernel has a smooth and infinitely differentiable function, thereby being used in this study.

For the Universal Kriging (UK), the mean value of the prediction result for the input point $x^{*}$ is shown below.

(7) $$\hat{y}(x^{*})=\mu_{\hat{y}}(x^{*})=f^{T}(x^{*})\hat{\beta }+r^{T}(x^{*})R^{-1}(y-F\hat{\beta })$$

where $\hat{\beta }$ is to compute the least squares estimate of the regression coefficients β.

(8) $$\hat{\beta }={\left(F^{T}R^{-1}F\right)}^{-1}F^{T}R^{-1}y$$

The variance of the predictor $\hat{y}(x^{*})$ is shown below.

(9) $$\begin{array}{cc} \sigma_{\hat{y}}^{2}(x^{*}) & =\sigma^{2}\left[1-\left[\begin{array}{cc} f^{T}(x^{*}) & r^{T}(x^{*}) \end{array}\right]{\left[\begin{array}{cc} 0 & F^{T}\\ F & R \end{array}\right]}^{-1}\left[\begin{array}{c} f(x^{*})\\ r(x^{*}) \end{array}\right]\right]\\ & =\sigma^{2}\left[1-r^{T}(x^{*})R^{-1}r(x^{*})+{\left(F^{T}R^{-1}r(x^{*})-f(x^{*})\right)}^{T}{\left(F^{T}R^{-1}F\right)}^{-1}\left(F^{T}R^{-1}r(x^{*})-f(x^{*})\right)\right] \end{array}$$

A special case of UK is the Ordinary Kriging (OK). The trend is an unknown constant $\beta_{0}$.

(10) $$f^{T}(x)\beta =\beta_{0}$$

(11) $${\hat{\beta }}_{0}=\frac{1_{m}^{T}R^{-1}y}{1_{m}^{T}R^{-1}1_{m}}$$

where $1_{m}$ is an m-length column vector of ones. The mean and variance values of the prediction results for the input point $x^{*}$ for OK can be written as the following equations.

(12) $$\mu_{\hat{y},OK}(x^{*})={\hat{\beta }}_{0}+r^{T}(x^{*})R^{-1}(y-1_{m}{\hat{\beta }}_{0})$$

(13) $$\sigma_{\hat{y},OK}^{2}(x^{*})=\sigma^{2}\left[1-r^{T}(x^{*})R^{-1}r(x^{*})+\frac{{\left[1-1_{m}^{T}R^{-1}r(x^{*})\right]}^{2}}{1_{m}^{T}R^{-1}1_{m}}\right]$$

In the Simple Kriging (SK), the trend is a known constant value μ shown below.

(14) $$\mu_{\hat{y},SK}(x^{*})=\mu +r^{T}(x^{*})R^{-1}(y-1_{m}\mu )$$

(15) $$\sigma_{\hat{y},SK}^{2}(x^{*})=\sigma^{2}\left[1-r^{T}(x^{*})R^{-1}r(x^{*})\right]$$

### 2.3. Latin Hypercube Sampling Method

The LHS method is one kind of advanced Monte Carlo simulations (MCS). By dividing the range of each variable into disjointed intervals with equal probabilities, the samples are randomly selected from each interval in LHS. It improves the stability of MCS and keeps satisfied accuracy and good convergence [18,19]. Consider a statistic system described by the function below.

(16) $$Y=F(X)\;X=\left\{X_{1},\;X_{2},\cdots ,X_{n}\right\}$$

where X is the random vector, and represents the independent input random variables. F is the operator, which performs a computer simulation, such as the finite element computation.

The LHS method divides the range of each vector component into disjointed subsets with equal probabilities. Samples of each vector component are captured from the respective subsets, according to Equation (17).

(17) $$x_{ki}^{j}=P_{X_{i}}^{-1}(U_{ij})$$

where i=1, ⋯, n; j=1, ⋯, m. n refers to the total number of vector components or dimensions of vector, and m is the number of subsets in a design. k is the subscript denoting a specific sample. P is the cumulative distribution function.

## 3. Program Implementation

**Figure 2:**
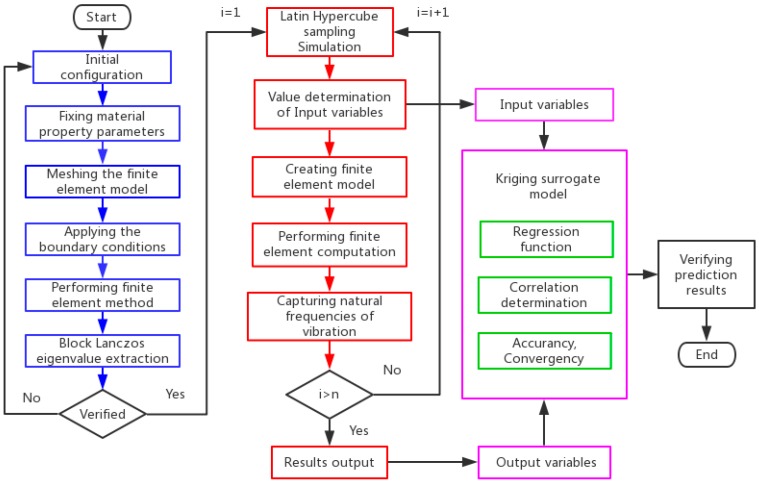
The flowchart of the Kriging surrogate model.

In order to demonstrate the KSM for graphene sheets, Figure 2 depicts the flowchart of KSM, which can be clearly concluded in blocks distinguished by different colors.

The blue boxes represent the procedure of the deterministic finite element model for vibration analysis of graphene sheets. First, the geometrical configuration of graphene sheets is defined. The corresponding parameters of bonds’ length and sectional diameter, as well as the height and width of a hexagonal 2D lattice are settled. Next, material parameters, which include the Young’s module, Poisson ratio, and physical density are provided. Then, perform the finite element model to compute the vibration modes and related natural frequencies.

After validation of the finite element model, the loop performs until sufficient times of sampling are completed, as presented in the red boxes. In the continuous loop, parameters corresponding to geometrical and material properties are the input variables of KSM, while the natural frequencies of the free vibration by finite element analysis are also captured and transferred to the procedure of KSM. These two groups of databases form sampling pairs in KSM.

In the pink boxes, the input variables of LHS and the output results of the finite element method (FEM) are sample pairs in KSM. In order to obtain satisfied prediction accuracy and good convergence, regression and correlation functions are required. The prediction results of KSM can be confirmed by comparison with the results in the reported references.

The numerical simulation model of graphene sheets was created by the ANSYS Parameter Design Language (Version 14.5, APDL, ANSYS, Cannonsburg, PA, USA). Carbon atoms in graphene are bonded together with covalent bonds, which form a hexagonal 2D lattice. The displacement of individual atoms under an external force is constrained by the bonds. For the modeling of the C-C bonds, the 3D elastic BEAM188 element was used. Each node has six degrees of freedom. The translations and rotations around the x, y, and z directions are included. In Table 1, the corresponding parameters, which are related to the geometrical and material properties, are listed. The interval ranges of material and geometrical parameters are settled, according to the relevant experimental and numerical data in the lierature [8,48,49]. The exact values of the parameters are replaced by the corresponding wide intervals.

## 4. Discussion and Results

In the deterministic beam finite element model (FEM), there are 6226 beams with 16,664 nodes. By LHS, the stochastic values for each parameter in the certain intervals are sampled for the finite element model of graphene sheets.

### 4.1. Statistical Results

**Figure 3:**
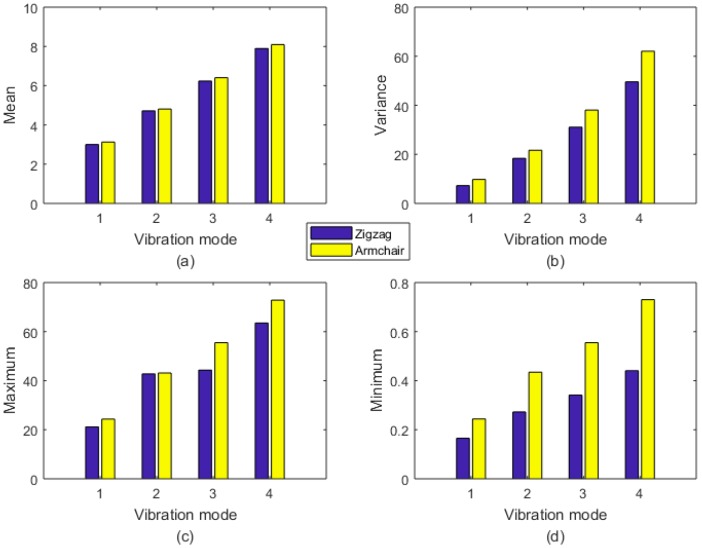
Probability results of LHS in a finite element model of graphene (**a**) is mean values, (**b**) is variance values, (**c**) is maximum values, and (**d**) is minimum values. The unit for the natural frequency is THz.

**Figure 4:**
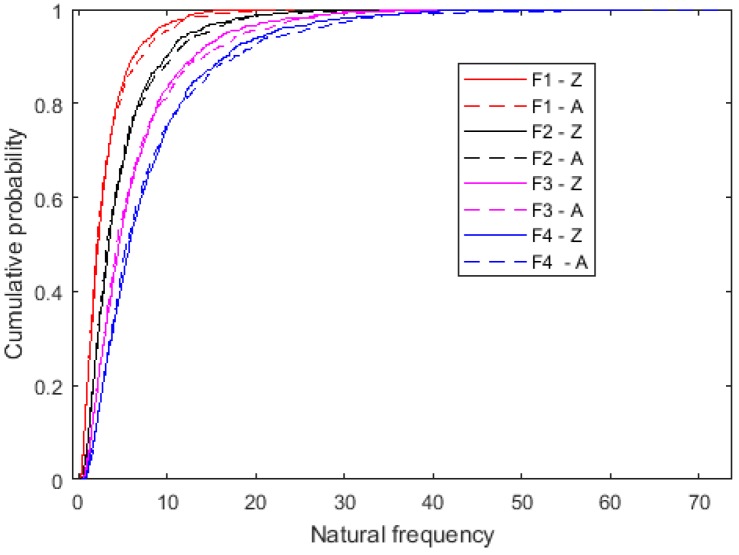
Cumulative probability of natural frequencies (the unit for the natural frequency is THz).

**Figure 5:**
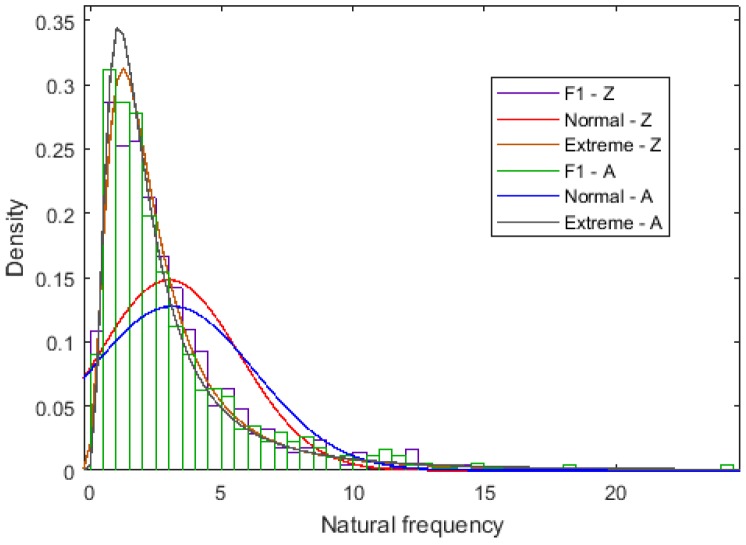
Probability density distribution for the first order natural frequencies (the unit for the natural frequency is THz).

As mentioned above, the uncertainties in the geometrical and material properties are propagated in the finite element model by LHS. Furthermore, 1000 samples for each input parameter and output result are obtained in the computational process. Based on the original database, the results are studied by the stochastic mathematics and probability theory, as shown in Table 2, Figure 3, Figure 4 and Figure 5.

In Table 2 and Figure 3, the differences between the Zigzag and Armchair graphene sheets are observed. In general, the mean values of the first four order natural frequencies are approximated in the Zigzag and Armchair graphene sheets. However, Armchair graphene sheets have larger values in the variance with maximum and minimum values from the respective database. Especially in the mimimum extreme condition, the results of Armchair graphene sheets are evidently larger than that of the Zigzag type. On one hand, the difference in the geometrical stuctures of the Armchair and Zigzag graphene sheets causes divergence in the statistical results. On the other hand, the stochastic sampling process can also lead to the deviation in the database of natural frequencies of Armchair and Zigzag graphene sheets. Furthermore, the differences between the zigzag and armchair edges in natural frequencies are also caused by the boundary conditions. Even though the micro structures of the zigzag and armchair graphene have only 90 degree rotation discrepancy, the boundary condition differences in the zigzag and armchair edges cannot be ignored. The six degrees of freedom (displacement in x, y, and z axes and rotation around to the x, y, and z axes) for atoms in the edges of graphene are supposed to be zero. The differences in zigzag and armchair edges lead to the inequality in natural frequencies.

The cumulative probability of the first four order natural frequencies is compared in Figure 4. For each order free vibration, the cumulative probability of the Zigzag and Armchair is close to each other. However, for all the first four order natural frequencies both in Zigzag and Armchair graphene sheets, the drag section in the large probability approching to one is very long and not negligible. This phenomenon is also presented in the results of the probability density distribution in Figure 5.

Even though all the input variables (parameters corresponding to the geometrical and material properties) of LHS are uniformly distributed in the sample space, the output results (natural frequencies of graphene sheets) do not obey the uniform or strict normal distribution, as shown in Figure 5. The drag section is an unevitable component in all ranges of natural frequencies. By taking comprehensive uncertainties in graphene sheets into consideration, the shape of probability distribution of natural frequencies is the peak shape with a long drag, while the natural frequencies are the ratio of stiffness matrices and mass matrices. It is reasonable to explain that the test equivalent results are concentrated in the narrow intervals with the possibility of large extreme values.

### 4.2. Comparison and Discussion

**Figure 6:**
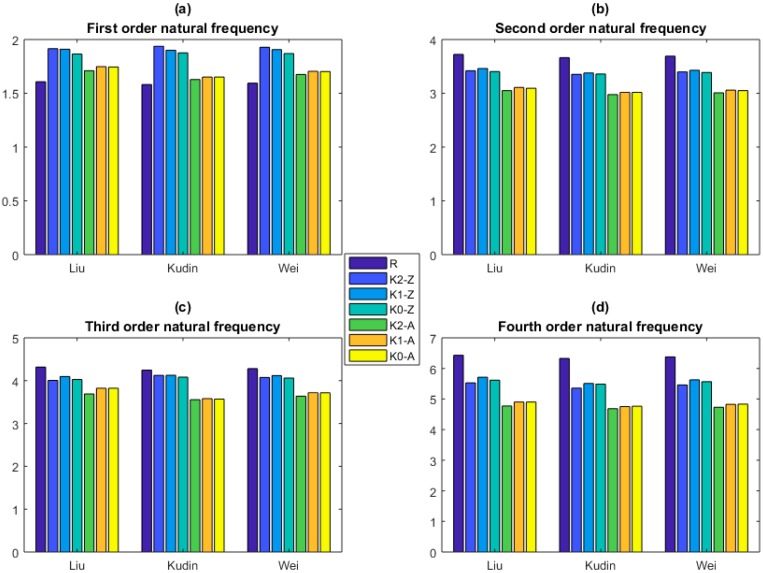
Comparison between the prediction results of KSM and that of DFT (the unit for the natural frequency is THz. K0–K2 are for the constant, linear, and quadratic functions. The Z and A are for Zigzag and Armchair graphene, respectively. (**a**), (**b**), (**c**) and (**d**) are the first four order natural frequencies, respectively.).

**Figure 7:**
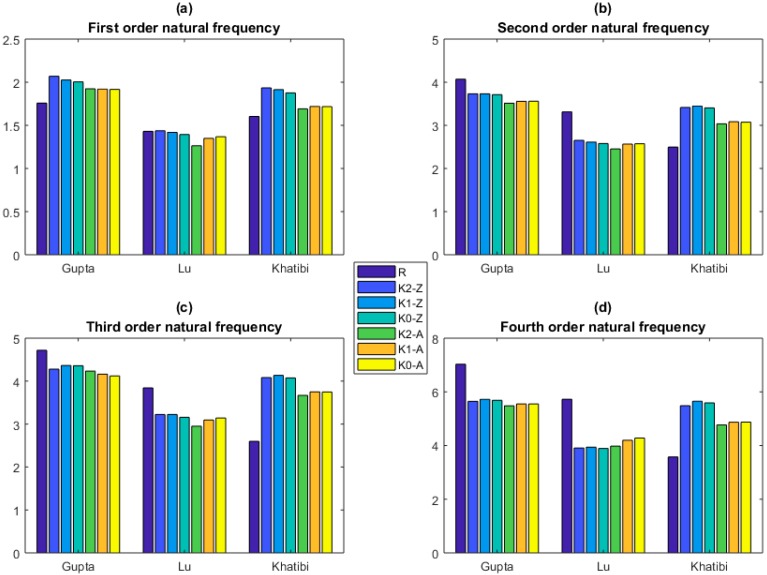
Comparison between the prediction results of KSM and that of MD (the units for the natural frequency is THz. K0–K2 are for constant, linear, and quadratic functions. Z and A are for Zigzag and Armchair graphene sheets, respectively. (**a**), (**b**), (**c**) and (**d**) are the first four order natural frequencies, respectively.).

**Figure 8:**
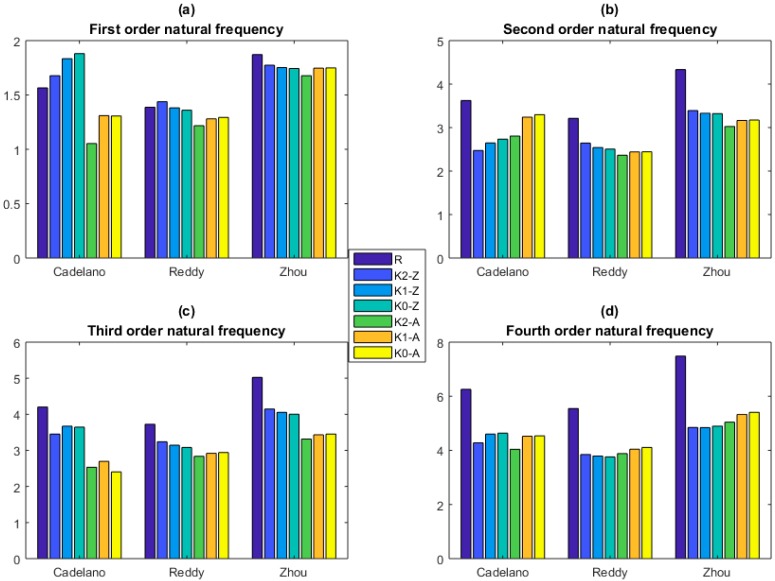
Comparison between the prediction results of KSM and that of MM (the unit for the natural frequency is THz. K0–K2 are for the constant, linear, and quadratic functions. Z and A are for Zigzag and Armchair graphene sheets, respectively. (**a**), (**b**), (**c**) and (**d**) are the first four order natural frequencies, respectively.).

The KSM is applied to predict the natural frequencies of graphene sheets. Comparison between the prediction results of KSM and that of the reported References [41,42,43,44,45,46,47,48,49] is demonstrated in Figure 6, Figure 7 and Figure 8.

In Figure 6, the prediction results of KSM are compared with that of Liu [38], Kudin [39], and Wei [40]. In the first order natural frequency, the prediction results of KSM is larger than the results of Liu [38], Kudin [39], and Wei [40]. The Armchair graphene sheets have the closer results to the reported reference [41,42,43,44,45,46]. In the second order, the predicted results are smaller than the reported results. The Zigzag graphene sheets have the more approximate results. The prediction results of Zigzag graphene sheets have a satisfied agreement with the reported results in the third order natural frequency. In the fourth order natural frequency, the Zigzag type graphen sheets have more consistent results. In addition, in the first four order natural frequencies, the prediction results of Zigzag graphene sheets are all larger than the Armchair graphene sheets. Furthermore, in order to fit the the relationship between the natural frequencies and parameters corresponding to the geometrical and material properties, the constant, linear, and quadratic functions in KSM are applied and compared in Figure 6. Besides the accuracy, the saitisfied convergence is reached in different orders of KSM.

The prediction results of KSM are all larger than that of Sadeghzadeh [43] in the first four order natural frequencies of graphene sheets, as shown in Figure 7. Moreover, the Zigzag graphene sheets have larger natural frequencies than Armchair graphene sheets. However, when the parameters corresponding to the geometrical and material properties take the same values of Gupta [41] and Lu [42], the difference between Zigzag and Armchair graphene sheets is not evident. In addition, the prediction results of KSM in the first order is larger than that of Gupta [41], but smaller in the other three order natural frequencies. The prediction results of KSM in the first order natural frequency is close to the results of Lu [42], especially in the Zigzag graphene sheets. However, the prediction results of KSM are smaller than that of Lu [42] in the other three order natural frequencies. In Figure 7, the convergence of KSM in distinct orders is also proven.

In Figure 8, for the first order natural frequency, the prediction results of KSM have appropriate accuracy with that of Reddy [45] and Zhou [46]. An evident deviation in the prediction results of KSM with that of Cadelano [44] is observed in the first four order natural frequencies. In addition, the prediction results are smaller than that of Reddy [45] and Zhou [46]. The difference of the Zigzag and Armchair is not negligible in the situation of Reddy [45] and Zhou [46]. Even though there is deviation between the prediction results of KSM and that of Reddy [45], Zhou [46], and Cadelano [44], the convergence in different orders of KSM demonstrates the robustness and reliability of KSM.

In addition, the results in this study are in good agreement with that of nonlocal plate model proposed by Ansari [50]. Different from the Timoshenko beam finite element model, the Mindlin plane equations are coupled with van der Waals interaction based on the nonlocal constitutive elastic equations. However, the out of plane behavior of graphene in the beam finite element model is larger than the results in the real situation due to the bond angle bending interaction. In addition, the geometrical parameters, such as the diameters, chiral angles, and length of carbon bonds, have the crucial influences on the axial and shear deformation copuling behaviors [51] and buckling critical stress [52]. More appropriate theoretical models for the mechanical properties’ analysis of gaphene are necessary to explore [53].

### 4.3. Uncertainty Analysis

The competitive predicition competence in KSM is not only applied in deterministic models, but can be used in uncertainty analysis. Based on the satisfied accuracy and robust convergence, KSM is applied in the uncertainty analysis of geometrical and material properties in graphene sheets.

**Figure 9:**
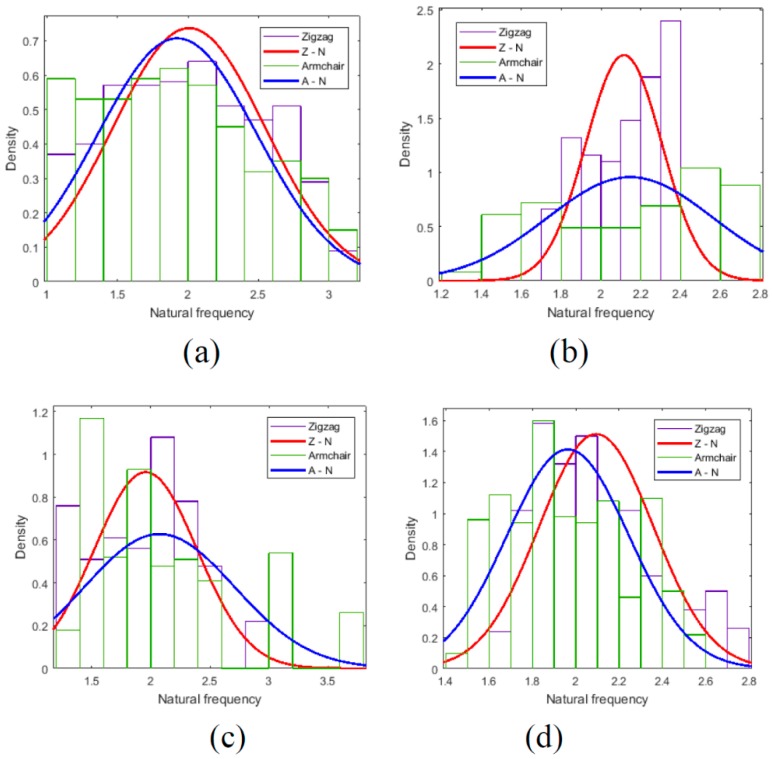
Probability density distribution of natural frequency for uncertainty analysis (**a**) is for *Bz* and *Ba*, (**b**) is for *Dz* and *Da*, (**c**) is for *Wz* and *Wa*, (**d**) is for *Hz* and *Ha*, and the unit for the natural frequency is THz.

**Figure 10:**
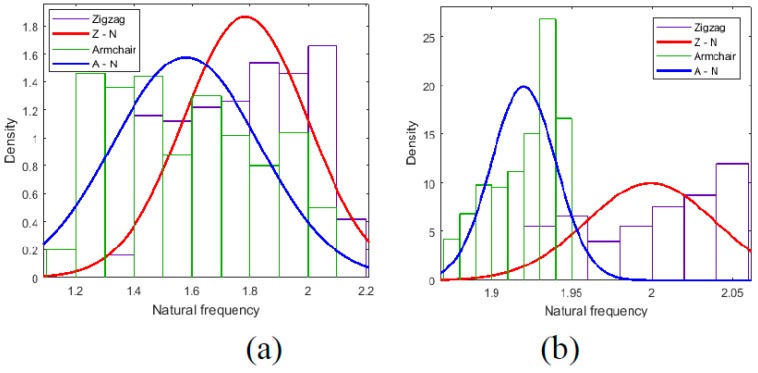

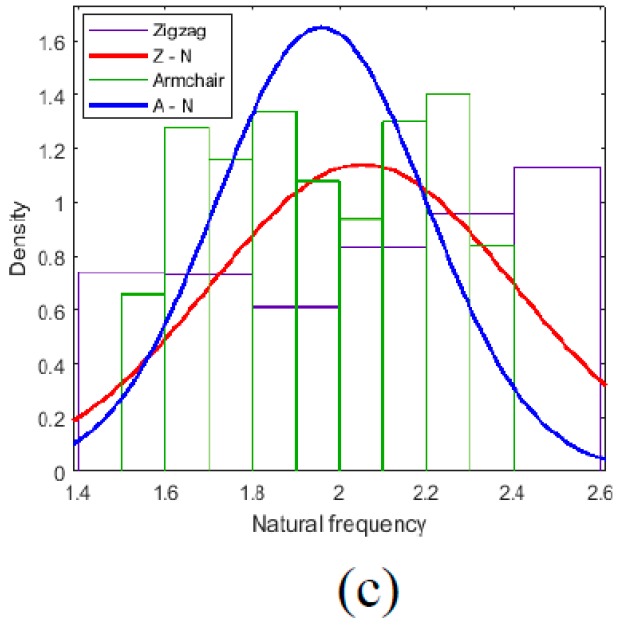
Probability density distribution of natural frequency for uncertainty analysis (**a**) is for *Ez* and *Ea*, (**b**) is for *Rz* and *Ra*, (**c**) is for *Tz* and *Ta*. The unit for the natural frequency is THz.

Table 3 and Table 4 list the results of uncertainty analysis of geometrical and material properties, respectively. When the corresponding parameters are sampled following an uniform distribution from the specific interval ranges, the probability density distributions of the first order natural frequency are demonstrated in Figure 9 and Figure 10.

In Figure 9a, when the length of bonds in graphene sheets is uncertain, the probability density distribution of Zigzag and Armchair graphene sheets is contiguous. The situation is analogous for the number of hexogons in height, as presented in Figure 10d. However, for the diameter of bonds’ section and the number of hexagons in width, the difference of the probability density distribution between Zigzag and Armchair graphene sheets is apparent. In general, the uncertainties in the diameter of bonds’ section and the number of hexagons in width leads to the lower and more gentle probability distribution in the Armchair than that in the Zigzag graphene sheets, as shown in Figure 9b,c. The Zigzag gaphene sheets have a more concentrated and peaky probability density distribution in the more narrow result interval. In Table 3, the values of variance including the maximum and minimum values also confirm this point. In a sense, Zigzag graphene sheets are more robust and less senstive to the uncertainties in the diameter of the bonds’ section and the number of hexagons in width than Armchair graphene sheets.

In addition, for the uncertainties in material properties in Figure 10, the probability density distribution of Zigzag graphene sheets is in the right side of Armchair graphene sheets, especially when the Poisson ratio is random. In Table 4, the mean values of the first order natural frequency in Zigzag graphene sheets are all larger than that of Armchair graphene sheets when the Young’s modulus, poisson ratio, or mass density is stochastic and uncertain. The variances of Zigzag graphene sheets are also larger than that of Armchair graphene sheets. In the free vibration, the uncertainties in material properties lead to more evident fluctuation in Zigzag graphene sheets.

Compared with the finite element method compuation, the advantage of time-saving in the KSM is presented in the uncertainty analysis process of graphene sheets. The time costs of natural frequencies and the vibration mode calcuation for each deterministic sample of graphene by finite element methods are nearly 5.6 s. However, based on the original database of LHS from the finite element model of graphene, the KSM can be applied to predict the natural frequencies with high efficiency. For the predicition of 1000 samples of graphene, it only takes approximated 12.3 s by KSM. When the parameters corresponding to geometrical and material properties are randomly distributed, performing the finite element model for sufficient times are computationally expensive and time consuming. Thus, the advantage of time-saving in the Kriging surrogate model compared with the traditional finite element model is evident.

In addition, even though the exact mathematical relationship or functional expression between the graphene size and natural frequencies are not explicit, the KSM is proposed to effectively describe the implicit relationship. Furthermore, the KSM is compatible for the database of the numerical simulation and experimental tests. It is possible to combine the results of numerical simulation and physical experiments and create a more inclusive and smart database. Therefore, developing the KSM in the study of graphene sheets and related nanomaterials are promising and crucial.

## 5. Conclusions

In summary, the Kriging surrogate model is not only a powerful method with satisfied accuracy and convergence in prediction, but also convenient and time-saving in uncertainty analysis of graphene sheets. The difficulties in uncertainty and vibration analysis of graphene sheets are well overcome by the Kriging surrogate model based on the LHS. With the comprehensive uncertainties in geometrical and material properties, the probabilty results of the LHS illustrate that the concentrated narrow interval with a long drag section is more appropriate than the strict normal probability density distribution. Furthermore, Zigzag graphene sheets are more robust and less senstive to the uncertainties of the geometrical property than Armchair graphene sheets in the free vibration. However, the uncertainties in materical properties cause larger fluctuation in Zigzag graphene sheets than in Armchair graphene sheets.

## Figures and Tables

**Table 1:** Geometrical and material parameters for graphene sheets *.

	Definition	Interval	Units
*B_z_*	The length of bonds in the Zigzag type	0.15–0.4	nm
*Ba*	The length of bonds in the Armchair type	0.15–0.4	nm
*Dz*	The diameter of bonds’ section in the Zigzag type	0.02–0.05	nm
*Da*	The diameter of bonds’ section in the Armchair type	0.02–0.05	nm
*Wz*	The number of hexagons in width in the Zigzag type	6–20	/
*Wa*	The number of hexagons in width in the Armchair type	6–20	/
*Hz*	The number of hexagons in height in the Zigzag type	20–60	/
*Ha*	The number of hexagons in height in the Armchair type	20–60	/
*Ez*	Young’s modulus of graphene sheets in the Zigzag type	0.2–2	TPa
*Ea*	Young’s modulus of graphene sheets in the Armchair type	0.2–2	TPa
*Rz*	Poisson ratio of graphene sheets in the Zigzag type	0.1–0.5	/
*Ra*	Poisson ratio of graphene sheets in the Armchair type	0.1–0.5	/
*Tz*	Physical density of graphene sheets in the Zigzag type	1500–4000	kg/m^3^
*Ta*	Physical density of graphene sheets in the Armchair type	1500–4000	kg/m^3^

* The way of probability distribution for each variable are uniform.

**Table 2:** Probability results of LHS in a finite element model of graphene *.

	Mean (THz)	Variance (THz^^2^)	Maximum (THz)	Minimum (THz)
*F*_1_-*Z*	3.0060	7.2567	21.1325	0.1654
*F*_2_-*Z*	4.7177	18.3757	42.7713	0.2730
*F*_3_-*Z*	6.2362	31.1114	44.2943	0.3413
*F*_4_-*Z*	7.8909	49.5804	63.4948	0.4411
*F*_1_-*A*	3.1309	9.7822	24.3613	0.2442
*F*_2_-*A*	4.8098	21.6774	43.1523	0.4345
*F*_3_-*A*	6.4075	38.0443	55.5136	0.5552
*F*_4_-*A*	8.0964	62.0176	72.8086	0.7306

* *F_1_-F_4_* represent the first-fourth natural frequencies. *Z* and *A* are for Zigzag and Armchair graphene sheets, respectively.

**Table 3:** Uncertainty analysis about parameters of geometrical properties.

	Interval	Mean (THz)	Variance (THz^^2^)	Maximum (THz)	Minimum (THz)
*Bz* (nm)	0.2–0.35	2.1151	0.0367	2.3469	1.7151
*Ba* (nm)	0.2–0.35	2.1472	0.1742	2.6792	1.3697
*Dz* (nm)	0.025–0.045	2.0073	0.2933	3.1165	1.0013
*Da* (nm)	0.025–0.045	1.9239	0.3184	3.0748	1.0522
*Wz*	8–18	1.9574	0.1890	2.9359	1.2455
*Wa*	8–18	2.0740	0.4036	3.7651	1.3056
*Hz*	30–50	2.0945	0.0695	2.7333	1.6979
*Ha*	30–50	1.9636	0.0796	2.5998	1.4943

**Table 4:** Uncertainty analysis about parameters of material properties.

	Interval	Mean (THz)	Variance (THz^^2^)	Maximum (THz)	Minimum (THz)
*Ez* (TPa)	0.6–1.3	1.7847	0.0457	2.1255	1.3774
*Ea* (TPa)	0.6–1.3	1.5794	0.0639	2.0562	1.1876
*Rz*	0.16–0.3	1.9990	0.0016	2.0442	1.9204
*Ra*	0.16–0.3	1.9200	0.0004	1.9421	1.8707
*Tz* (g/cm^3^)	1.6–3.6	2.0531	0.1223	2.5258	1.4059
*Ta* (g/cm^3^)	1.6–3.6	1.9587	0.0583	2.3490	1.5493

## Data Availability

The raw data required to reproduce these findings are available to download from [https://drive.google.com/drive/folders/1r4o8h7m6eQ7EDhCuTip5BCGK_MG1U3zn?usp=sharing].
